# Five-Year Experience of Interwoven Self-Expanding Stent Implantation in Stenotic Kinking of Below the Knee Prosthetic Bypasses

**DOI:** 10.1007/s00270-024-03728-7

**Published:** 2024-04-23

**Authors:** Szymon Salamaga, Michał-Goran Stanišić, Hubert Stępak, Maciej Błaszyk, Zbigniew Krasiński

**Affiliations:** 1 Department of Vascular and Endovascular Surgery, Angiology and Phlebology, Poznan University of Medical Sciences, Długa Street, 61-848 Poznan, Poland; 2 Department of Radiology, Poznan University of Medical Sciences, 1/2 Długa Street, 61-848 Poznan, Poland

**Keywords:** Chronic limb ischemia, Supera stent, Thrombosis, Below the knee prosthetic bypass, Bypass kinking

## Abstract

**Purpose:**

The purpose of this study was to evaluate the 5-year real-world results of Supera stent implantation in below the knee prosthetic bypasses (BKPBs). All the procedures were performed because of a history of recurrent thrombosis of the graft and significant stenotic kinking of the prosthesis during knee flexion. A Supera stent was implanted to prevent the next potential BKPB thrombosis.

**Materials and Methods:**

Fourteen patients were included in this single-center, retrospective observational cohort study. All patients underwent Supera stent implantation in infrainguinal prosthetic bypass between 2012 and 2017, due to a history of recurrent thrombosis and kinking of the prosthetic bypass.

**Results:**

Prior to Supera stent implantation procedure, all the patients had more than one episode of acute limb ischemia caused by thrombosis of the BKPB. The median number of BKPB thromboses prior to Supera stent implantation was 3 and ranged from 2 to 6. Technical success was achieved in all cases. Primary patency rates at 12, 24, 36 and 60 months were 71.4%, 57.1%, 57.1% and 14.3%, respectively. Secondary patency rates at 12, 24, 36 and 60 months were 78.6%, 64.3%, 64.3% and 35.7%, respectively. One stent fracture was reported during 60-month follow-up. Major amputation was performed in 6 patients in 5-year follow-up.

**Conclusion:**

Supera stent in treatment of recurrent thrombosis of BKBP is a safe procedure with acceptable mid-term results. However, larger and comparable prospective studies are needed for broader analysis of this procedure.

**Graphical Abstract:**

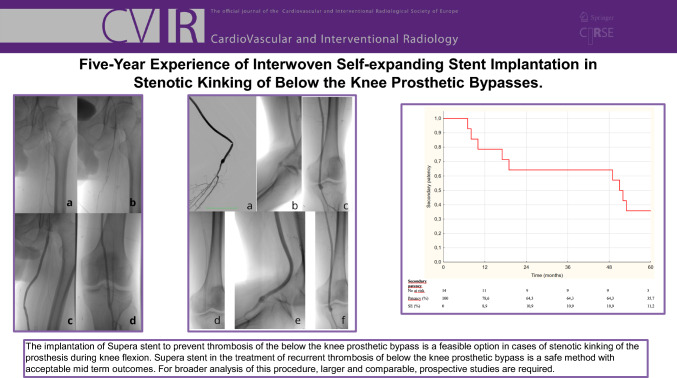

## Introduction

Failed infrainguinal bypass is associated with a poor limb prognosis, especially in cases of procedures that were initially performed for limb salvage [1]. Graft thrombosis in most cases is provoked by inadequate inflow, poor runoff vessels, significant anastomotic stenosis, or hypercoagulable state [2]. One of the reasons for reduced patency rates is stenotic kinking of the graft during knee flexion [3, 4]. Stenotic kinking is an underdiagnosed cause of graft occlusion. Diagnosis should be made by measuring the ankle brachial index (ABI) in flexion and extension of the knee joint, followed by duplex ultrasound and angiography in two perpendicular projections with the knee in flexion and extension. Treatment of stenotic kinking of the below the knee prosthetic bypass (BKPB) was previously limited to surgical revision with implantation of a new prosthetic graft [3, 5].

The Supera stent (Abbott Vascular, Santa Clara, Calif, USA) is a self-expanding, braided, interwoven nitinol stent with a helical construction. The Supera stent has high radial force and is therefore able to withstand increased compression and biomechanical stress [6–8].

The aim of this study was to evaluate the 5-year results of Supera stent implantation in BKPB due to recurrent graft thrombosis and stenotic kinking of the prosthesis.

## Materials and Methods

This is a single-center, observational cohort study with a retrospective evaluation of the acquired data. All patients underwent Supera stent implantation in an infrainguinal polytetrafluoroethylene (PTFE) prosthetic bypass between 2012 and 2017. Inclusion criteria were: implantation of the Supera stent to the femoropopliteal below the knee prosthetic bypass (BKPB), history of recurrent graft thrombosis, proper outflow and inflow from the prosthesis, and stenotic kinking during knee flexion, and no other possible explanation for recurrent graft thrombosis. Exclusion criteria were implantation of the Supera stent in femoropopliteal bypass due to anastomotic stenosis. The study was conducted in accordance with the Helsinki Declaration. Institutional bioethics committee approval was obtained.

### Procedural Details

Prior to Supera stent implantation procedure, all the patients had more than one episode of thrombosis of the BKPB. Episodes of graft thrombosis were successfully treated with targeted catheter-directed thrombolysis prior to the Supera stent implantation. In some cases, thrombolysis was combined with an additional procedure. The Supera stent was implanted to prevent recurrent graft thrombosis. This procedure was scheduled for the next hospitalization. Patients were eligible for Supera stent implantation based on the following criteria: history of recurrent graft thrombosis, proper outflow and inflow from the prosthesis as demonstrated by Duplex ultrasound and computed tomography angiography (CTA), hemodynamically significant stenotic kinking during knee flexion as demonstrated by duplex ultrasound and digital subtraction angiography (DSA), and no other possible explanation for recurrent graft thrombosis. Duplex ultrasound and CTA were performed before the procedure to evaluate the inflow and outflow of the BKPB. BKPB were patented at the time of stent implantation. DSA was performed in 90° flexion and full extension of the knee joint. If angiography revealed a stenotic kinking during knee flexion, a Supera stent was implanted at the site of the stenosis. After implantation, angiography was performed with the knee joint in 90° flexion and full extension. The diameter of the Supera stent used was not in a 1:1 ratio to the diameter of the graft. Based on the previous multiple episodes of graft thrombosis, it was suspected that mural thrombi were present in the graft. To match the Supera stent diameter to the graft lumen in a 1:1 ratio with mural thrombi, a 5.5-mm Supera was implanted in the 6-mm graft and a 7.5-mm Supera was implanted in the 8 mm graft.

### Definitions

The definition of major adverse cardiovascular events (MACE) included: acute coronary syndrome, stroke (either ischemic or hemorrhagic stroke), cardiovascular death and all-cause death. Major adverse limb events (MALE) were defined as either major amputation of the revascularized limb or reintervention of the revascularized segment. Primary patency was defined as no need for target limb revascularization based on clinical indication or stenosis on follow-up duplex ultrasound. Secondary patency was defined as the cumulative patency rate including reintervention after occlusion. Technical success was defined as the achievement of vessel patency with less than 30% residual stenosis.

### Follow-up

Complications during the 30-day post-procedure period were evaluated. Patients were reevaluated at clinic visits 1 month, 6 months, 12 months, and then every 12 months after the procedure. At the follow-up visits, patients underwent physical examination and duplex ultrasonography. Duplex ultrasound was performed with 90° of knee joint flexion and full knee joint extension. All patients were prescribed dual antiplatelet therapy (aspirin + clopidogrel) for at least 6 months.

### Statistical Analysis

Continuous variables were reported as mean ± standard deviation or as median (range). Categorical variables were reported as counts (percentages). Kaplan–Meier analysis was used to determine patency rates. Statistical significance was confirmed with a *p* value < 0.05. Statistical analysis was performed with Statsoft Statistica 13.3 software (TIBCO Software Inc., Palo Alto, CA, USA, 2017).

## Results

Fourteen patients were included in the study. Seven BKPBs (50%) were performed with Miller cuff modification of the distal anastomosis. Patients characteristics presenting symptoms and indications for initial BKPB surgery are summarized in Table 1.

**Table 1:** Baseline patients demographic and clinical characteristics

Characteristics	Number of patients *n* = 14	Percentage or range
Male gender	12	85.7%
Age (years) (median)	60.5	17 (IQR)
BMI (kg/m2)	28.0 ± 3.0	24.2–33.2
Arterial hypertension	12	85.7%
Smoking	10	71.4%
Diabetes mellitus	8	57.1%
Hyperlipidemia	9	64.3%
Cerebrovascular disease	2	14.3%
Coronary artery disease	9	64.3%
Renal insufficiency requiring hemodialysis	0	0%
Rutherford classification (prior initial BKPB surgery)		
2	0	0%
3	1	7.1%
4	2	14.3%
5	3	21.4%
6	1	7.1%
CLTI (indication for BKPB)	6	42.9%
ALI (indication for BKPB)	7	50.0%

*CLTI* Chronic limb-threatening ischemia, *BMI* Body mass index, BKPB Below the knee prosthetic bypass, *ALI* Acute limb ischemia

**Fig. 1:**
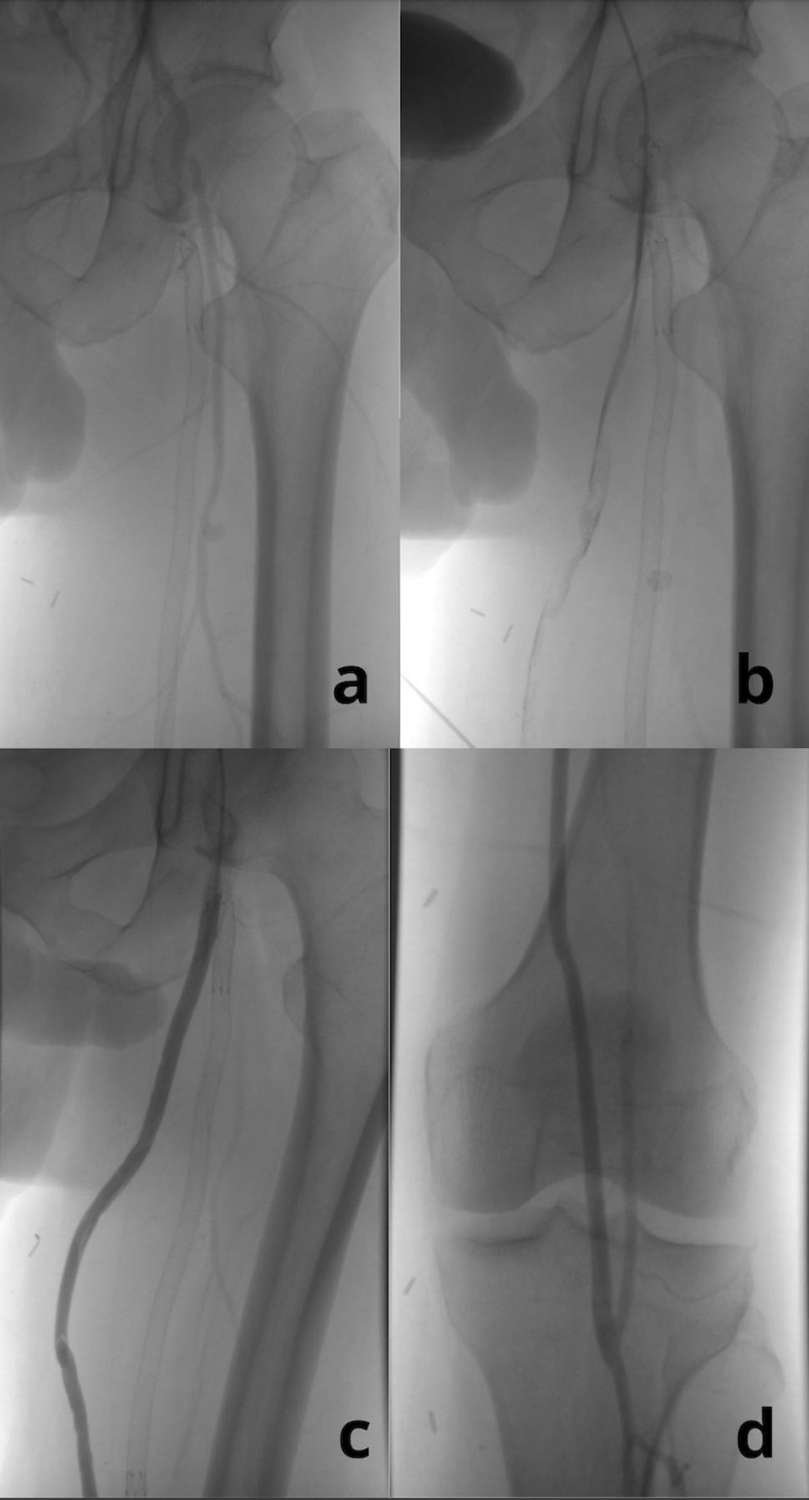
Thrombosis of the BKPB and results of catheter directed thrombolysis. **a** Thrombosis of the BKPB. Multiple occluded stents are visible in the superficial femoral artery (SFA). Stents in the SFA have been occluded before BKPB surgery. **b** Administration of the contrast directly to thrombosed BKPB. **c** and **d** Patent BKPB after 48 h of catheter directed thrombolysis

The median time from BKPB surgery to the analyzed Supera stent implantation procedure was 12 months. The median number of thromboses of the BKPB prior to Supera stent implantation was 3 and ranged from 2 to 6. The time interval between episodes of BKPB thrombosis ranged from 1 to 29 months with a median of 4 months. The time interval between recurrent thrombosis and Supera stent implantation ranged from 1 to 2 months, and no reocclusion was observed during the waiting time. Images of BKPB thrombosis before and after treatment are presented in Fig. 1.

**Fig. 2:**
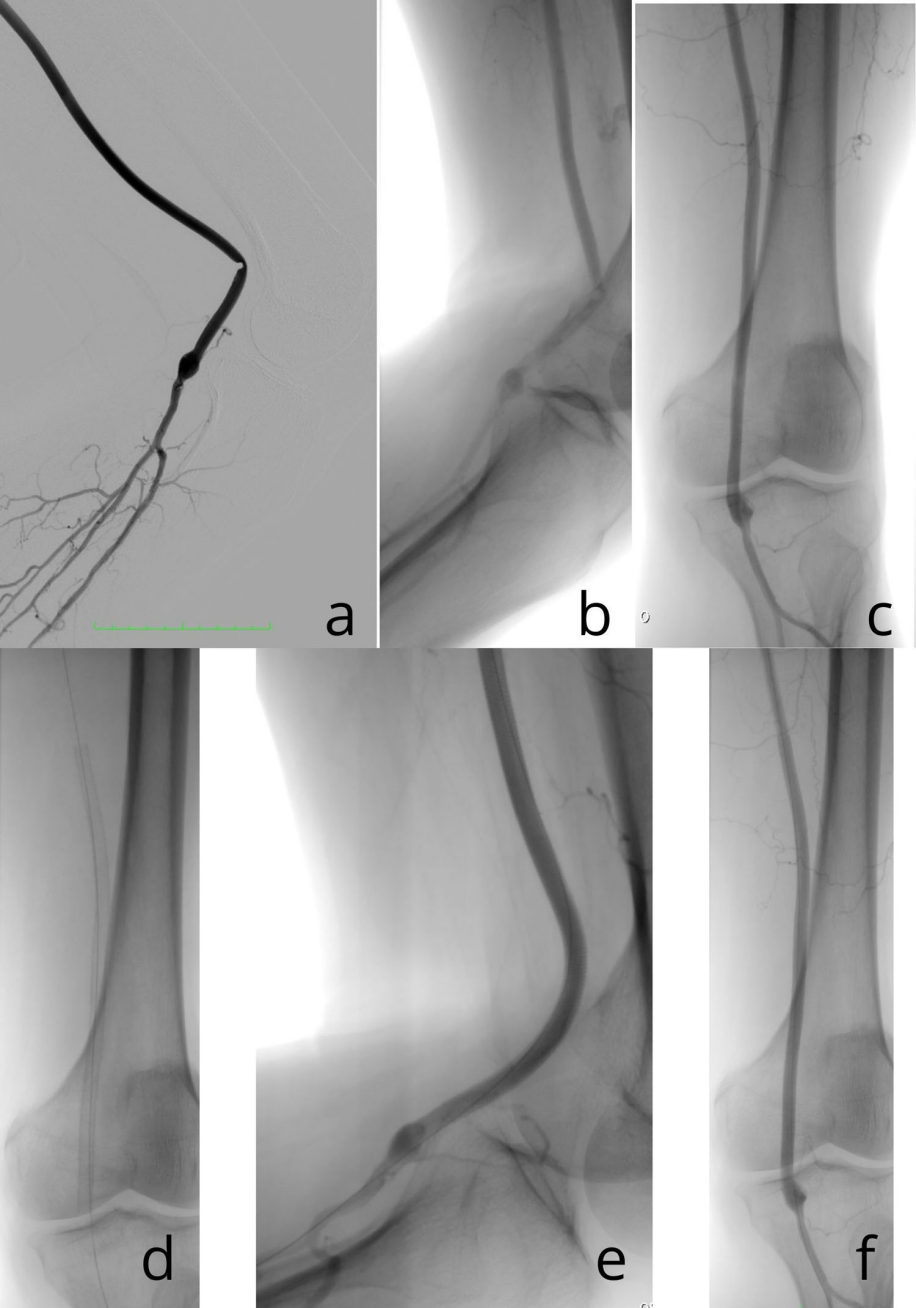
Implantation of the Supera stent in BKPB. BKPB has been patented for more than 40 months after Supera stent implantation. Before Supera stent implantation, two episodes of graft thrombosis occurred 9 months and 10 months after BKPB procedure. **a** and **b** Stenotic kinking of the prosthesis was observed during 90° knee flexion. **c** Stenosis of the BKPB was not visualized during knee extension. **d** Long 5.5 × 200 mm Supera stent was implanted in BKPB. **e** Angiography performed in 90° knee flexion. After Supera stent implantation, stenotic kinking of the BKPB was liquidated. **f** Angiography performed in full knee extension after Supera stent implantation

Stents characteristics are shown in Table 2. In all cases, only one stent was implanted. The median stent diameter was 5.5 mm. Technical success of Supera stent implantation was achieved in 100% of the procedures. Supera stent implantation in stenotic kinking of BKPB is illustrated in Fig. 2.

**Table 2:** Supera stents characteristics

Characteristics	Number	Percentage or range
Stent diameter		
5.5 mm	8	57.1%
7.5 mm	6	42.9%
Stent diameter (median) (mm)	5.5	5.5–7.5
Stent length		
100 mm	5	35.7%
150 mm	5	35.7%
200 mm	4	28.6%
Stent length (median) (mm)	150	100–200

No MACE or MALE were observed during the 30-day follow-up period. However, 1 minor complication was noted: access site hematoma.

**Fig. 3:**
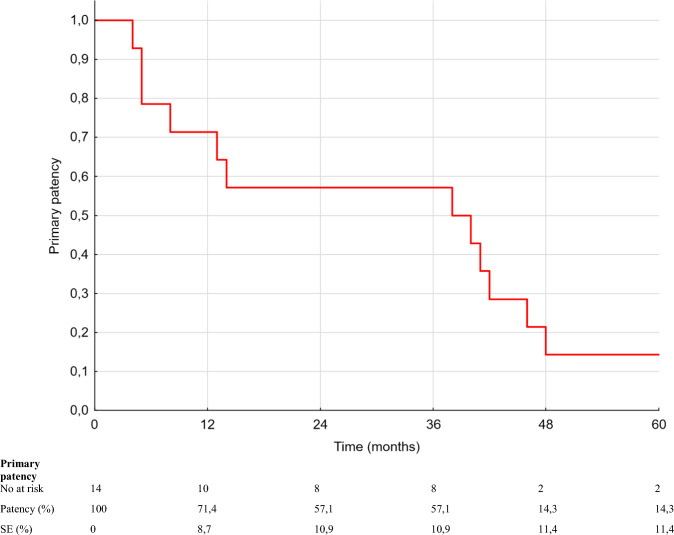
Kaplan–Meier estimates establishing primary patency rate of Supera stent implantation on duplex 5-year follow-up. Eight episodes of Supera stent thrombosis, and 4 episodes of restenosis of the Supera stent were observed of the BKPB failed in the first year after Supera stent implantation

**Fig. 4:**
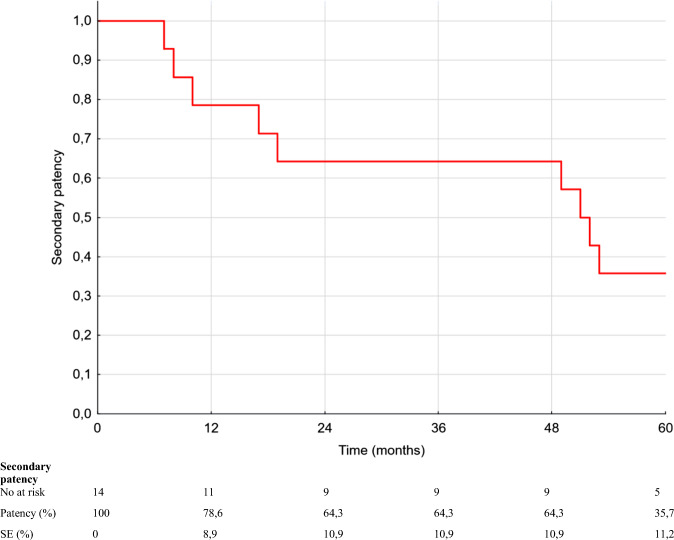
Kaplan–Meier estimates establishing secondary patency rate of Supera stent implantation on duplex 5-year follow-up. Secondary patency was defined as the cumulative patency rate including reintervention after occlusion

The median follow-up was 51 months and ranged from 7 to 108 months. However, 6 patients were lost to follow-up before 60 months. Two patients were lost to follow-up in the first year, three patients were lost to follow-up between 12 and 24 months, and one patient was lost to follow-up after 48 months. Four individuals lost to follow-up were patients who had undergone above-the-knee amputation. Primary patency rates at 12, 24, 36 and 60 months were 71.4%, 57.1%, 57.1% and 14.3%, respectively (Fig. 3). Secondary patency rates at 12, 24, 36 and 60 months were, respectively, 78.6%, 64.3%, 64.3% and 35.7%, respectively (Fig. 4). Episodes of Supera stent and graft thrombosis were treated with catheter-directed thrombolysis. Four cases of Supera stent restenosis were successfully treated with PTA. One stent fracture was observed during 60 months of follow-up. The stent fracture occurred 5 months after stent implantation, which resulted in BKPB thrombosis and above the knee amputation. All six amputations were associated with Supera stent and BKPB thrombosis and failure. Four amputations were above the knee amputations (AKA), and two were below the knee amputations (BKA).

## Discussion

The feasibility of Supera stent for bypass graft salvage has not been analyzed in the existing studies except for one case report [9]. The results of this small series suggest that Supera stent implantation should be considered an acceptable option for the treatment of stenotic kinking of BKPB. In follow-up, eight patients did not experience BKPB thrombosis during the first 24 months after Supera stent implantation. However, recurrent thrombosis occurred at short intervals prior to stent implantation. Nevertheless, limb salvage rate was 57.1% and number of secondary events was relatively high. Those aspects question the results of this study and may suggest the need for more precise selection of patients. Four patients experienced BKPB thrombosis in the first year after Supera stent implantation, suggesting that stenotic kinking was not the cause of BKPB thrombosis. Implantation of the Supera stent in the BKPB results in straightening of the bypass. This could potentially lead to kinking at the different levels and tension at anastomoses. Graft kinking at the different levels or tension at anastomoses may be the reason for early graft thrombosis after Supera stent implantation. Hematological disorders may also be the reason for early thrombosis of the BKPB graft after Supera stent implantation. Progression of arteriosclerotic disease may lead to graft thrombosis in mid- or long-term observation.

Intimal hyperplasia at the distal anastomosis is the most common etiology for the femoropopliteal bypass failure [10]. However, stenotic kinking of the prosthetic vascular graft has also been shown to be a significant risk factor for BKPB failure [3, 11]. Kinking of a BKPB during knee flexion may provoke thrombosis by two mechanisms. First, knee flexion may occlude the graft and, if prolonged, lead to thrombosis. The second mechanism relates to turbulence, jet phenomena and in the long-term neointimal proliferations provoked by kinking of the vascular graft. These lesions may be tears or ulcerations of the neointimal lining that promote local thrombosis or hyperplasic neointimal stenosis of the graft [11–13].

Stenotic kinking of the BKPB during knee flexion is a rare phenomenon, and most of the cases are treated surgically. Most of the literature focuses on the treatment of anastomotic stenosis [14–18]. Fujimura et al. analyzed the results of treatment of previously occluded above-the-knee infrainguinal bypasses by Viabahn stent-graft implantation [19]. Presumably, other vasculomimetic stents could be used instead of Supera stent to achieve similar results. The optimal time interval between Supera stent implantation for stenotic kinking of the graft and recurrent thrombosis was not determined in this study. Considering the short time intervals between episodes of recurrent graft thrombosis, we suggest stent implantation directly after treatment of BKPB thrombosis.

The main limitations of the study are: small cohort of patients, one type of stent used, retrospective design of the study and lack of randomization and comparison with different treatment methods.

## Conclusions

Implantation of the Supera stent to prevent thrombosis of the BKPB is a feasible option in cases of stenotic kinking of the prosthesis during knee flexion. Supera stent in the treatment of recurrent BKPB thrombosis is a safe procedure with acceptable mid-term results. Larger and comparable prospective studies are needed for a more comprehensive analysis of this procedure.
